# Demonstration of the FLASH Effect Within the Spread-out Bragg Peak After Abdominal Irradiation of Mice

**DOI:** 10.14338/IJPT-20-00095

**Published:** 2021-08-19

**Authors:** Tucker Evans, James Cooley, Miles Wagner, Tianning Yu, Townsend Zwart

**Affiliations:** 1 Mevion Medical Systems, Littleton, MA, USA

**Keywords:** FLASH, proton therapy, intensity modulated proton therapy

## Abstract

**Purpose:**

The effects of FLASH-level dose rates delivered at the spread-out Bragg peak (SOBP) on normal tissue damage in mice were investigated.

**Materials and Methods:**

Fifty nontumor-bearing mice received abdominal irradiation, 30 at FLASH dose rates (100 Gy/s) and 20 at conventional dose rates (0.1 Gy/s). Total dose values ranged from 10 to 19 Gy, delivered in a single spot by a synchrocyclotron proton therapy system. Centered on the abdomen, the collimated field delivered was an 11-mm diameter circle with a water-equivalent depth of 2.4 cm from entrance to distal 80% dose. A ridge filter was used to provide dose uniformity over the full 2.4-cm range. The spatial distribution was identical for both the FLASH and conventional deliveries.

**Results:**

Overall survival and individual mouse weights were tracked for 21 days after the exposure date, and LD50 values were compared for the FLASH and conventional dose rate groups. Mice exposed to FLASH dose rates had a higher LD50 value as compared with mice exposed to conventional dose rates, with a dose-dependent improvement in survivability of 10% to 20%. The FLASH cohort also showed greater or equal percent population survival for each day of the study.

**Conclusion:**

These results are preliminary confirmation of the potential for the combination of the advantages of the Bragg peak with the normal tissue sparing benefits of FLASH treatments. This experiment also confirms that pulsed synchrocyclotrons can be used for the purpose of FLASH research and treatment.

## Introduction

Research into the differential effect of high- and low-dose rates for radiotherapy has shown that higher dose rates (typically over 40 Gy/s) lead to improved normal tissue sparing. Multiple groups have demonstrated this effect using photons [1, 2], electrons [3–7], and protons [8–14]. Results from these experiments support the claim that higher dose rate treatments with total treatment time under 1 second exhibit less normal tissue toxicity while maintaining tumor control typical of conventional treatments. Many industry leaders suggest that FLASH is a revolutionary discovery in the field of radiotherapy, expanding applications of radiotherapy to previously contraindicated cases [15]. For eventual applications to proton therapy, it is important to consider whether the effects observed in these studies can be combined with the superior conformality offered by the Bragg peak. While proton machines can be used for shoot-through techniques, the benefits of FLASH irradiation would have to be quite high to outweigh the conformality of a standard proton therapy delivery that leverages the sharp drop-off and low entry dose of the Bragg peak. This study marks a first step toward combining the benefits of both the FLASH effect and intensity modulated proton therapy.

Our experiment follows Loo et al [16] who used a linear accelerator modified to deliver high-dose rate electron pulses in which mice exposed at FLASH dose rates showed significantly higher survival rates over the same range of doses and showed a statistically significant increase in LD50 value between FLASH and conventional deliveries. Treatments targeted the mouse abdomen, with mortality as the primary observable. The mice used in the study were nontumor bearing, indicating that any increase in survival corresponds to an improvement in normal tissue sparing that is not expected to come at any cost to tumor control. The synchrocyclotron (S250i; Mevion Medical Systems) used for this experiment delivered an average dose rate of 96 Gy/s at a depth of 1.2 cm, chosen to represent the middle of a mouse abdomen. This translates to a maximum dose rate of 120 Gy/s, and a corresponding minimum dose rate of 72 Gy/s at the edges of the delivered field. This is well above the generally accepted 40 Gy/s threshold for the FLASH effect.

## Materials and Methods

The cohort structure used in this study is shown in the **Table**. Two cohorts were used, a FLASH cohort and a conventional dose rate cohort, with respective average dose rates of 96 and 0.1 Gy/s. Target doses were evenly spaced across their respective ranges of 10 to 19 Gy in the case of the FLASH cohort and 10 to 16 Gy in the case of the conventional cohort. A slightly wider range of doses was used for the FLASH case to increase the likelihood of spanning the LD50, as the expected magnitude of the shift in LD50 value was uncertain. Previous testing yielded an approximate LD50 value for conventional irradiation of 13 to 14 Gy. We used this as the center of the conventional dose range. The FLASH range covered the same range as the conventional group, with an overage of 3 Gy at the top of the range to capture any upward shift in the LD50. The actual doses delivered, as measured with the EBT-XD film, are shown in **Figure 2**.

**Table.** Dose distributions and dose ranges of the FLASH and conventional mouse delivery cohorts.

**Cohort**	**Mice, n**	**Dose rate, avg. Gy/s**	**Dose range, Gy**
FLASH	30	96	10–19
Conventional	20	0.1	10–16

### Dosimetry

To assess the effect of FLASH dose rates within a spread-out Bragg peak (SOBP), it was necessary to develop dosimetry systems capable of accurately measuring both FLASH and conventional dose rates. We employed a combination of dosimetry techniques, both passive and active, to allow for redundancy and effective beam control. There is a large body of literature focusing on the characterization of dosimeter responses to ultra-high dose rate deliveries, including discussions of recombination effects in parallel plate ionization chambers [17–19] and film dose rate effects [20, 21]. We used a PPC05 chamber (IBA) as our primary calibration unit for recording total dose and dose rate at the delivery location in advance of live trials [22]. This allowed for calibration of the following 2 other detectors: a FLASH-capable transmission ionization chamber (or FLASH TIC) and EBT-XD films placed immediately upstream of the mice during the experiment. The FLASH TIC was used for control and the film used for absolute dosimetry.

The FLASH TIC was designed to have a comparable structure to the PPC05 detector, with a similar gap but larger area allowing for the entire field to be captured. In addition, the FLASH TIC has no electronics or other structures running across the beam line, and so it interferes minimally with the beam's internal distribution as it passes through. With a gap of 0.8 mm as compared with the 0.6-mm gap of the PPC05, the resulting recombination factor is similar and the detector performs linearly across the whole range of dose rates that were used over the course of these experiments. To avoid the uncertainty due to recombination effects and saturation of the electronics associated with the standard TICs, the FLASH TIC was tied into the dosimetry system of the S250i and used as the primary control for termination of the beam. Previous research and theory supports the claim that the effect of recombination for parallel-plate transmission ion chambers varies strongly with the gap between the parallel plates and the voltage applied [17–19]. The FLASH TIC used a bias voltage of 500 V, similar to the maximum bias voltage of the PPC05.

The EBT-XD film provided an independent absolute dose measurement after the conclusion of the experiment. One film was taken for every mouse that was irradiated. The dose response of the EBT-XD film and the other films of the same EBT line is independent of dose rate [20, 21]. The challenge of addressing such a wide range of dose rates is 2-fold. At high-dose rates, recombination and saturation are problematic and at low-dose rates, drift becomes a major issue, as even 1 pA of drift can represent a significant correction to the reported dose over the timescale of minutes required for conventional deliveries. We addressed both of these issues through the use of the FLASH TIC and the application of a drift correction for each of the units in use. The FLASH TIC was used only for dose control, not for the calculation of the total dose values used in fits for the survivorship curve. This was due to the fact that even with its small gap there was still a recombination effect on the order of 5.5% over the full range of delivery at high-dose rates. At low-dose rates, we observed drift effects on the FLASH TIC that also amounted to 5% to 6% of the value reported by the film. There was no measurable drift over the course of the short deliveries for the FLASH cohort. However, because the magnitude of the drift was comparable to that of the effect that we hoped to demonstrate, we felt that film was the more reliable measure of absolute dose.

### Beam parameters and dose rate control

We treated all the mice in the study in a single day, using a modified version of a clinical synchrocyclotron (S250i; Mevion Medical Systems). The beam was a single-scattered spot with an energy of 230 MeV. To deliver dose at the Bragg peak, we placed the mouse downstream of 30-cm water-equivalent thickness (WET) of boron carbide absorbing material and an 11-mm diameter brass aperture. In addition, a small amount (1.8-mm WET) of polyethylene absorbing material was used to slightly adjust the depth of the delivery. The FLASH assembly depicted in **Figure 2** allowed the mouse and absorbing material to be positioned at a configurable distance from the apparent source of the radiation and centered laterally. A ridge filter placed just upstream of the boron carbide absorber material modified the depth profile of the beam to create an SOBP without the need for an active range shifter assembly. The ridge filter consists of a grid of machined holes through an acrylic plastic (PMMA) block. The WET of the block sets the desired separation of the peaks in the final distribution. In addition to contributing to energy selection, the boron carbide downstream of the ridge filter also scattered the beam enough to make a more uniform field and to smooth out the profile immediately downstream of the ridge filter. The resulting 80% depth contour was at a depth of 2.4 cm. The full beam distribution downstream of the aperture is shown in **Figure 3**. The 2.4-cm width of the SOBP was confirmed in previous trials with the multilayer ionization chamber to be slightly greater than the WET of the mice used, indicating that the entire mouse is within the SOBP (ie, between the 80% contour lines). Alignment before testing placed the peak dose rate at the center of the collimator and was maintained throughout testing. We achieved an averaged dose rate of 96 Gy/s over a volume of 2 cm^3^. An 11-mm diameter brass aperture constrained the lateral profile.

The proton accelerator used is a pulsed synchrocyclotron, with a time between pulses of 1.3 ms. For this experiment, the duration of the ion source pulse was 21 μs. There were 80 pulses to achieve a 10 Gy dose at a rate of 100 Gy/s, corresponding to an instantaneous dose rate of 6200 Gy/s during the pulse for FLASH dose rates with a repetition rate of 756 Hz. This pulse structure was maintained for the low-dose rate deliveries, with an intrapulse dose rate of approximately 6.2 Gy/s. Using adjustments to the radiofrequency envelope and gas flow we detuned the cyclotron to provide a smaller amount of charge per pulse while maintaining identical spatial distributions and the same pulse timing.

### Mouse handling and anesthesia

**Figure 1.**
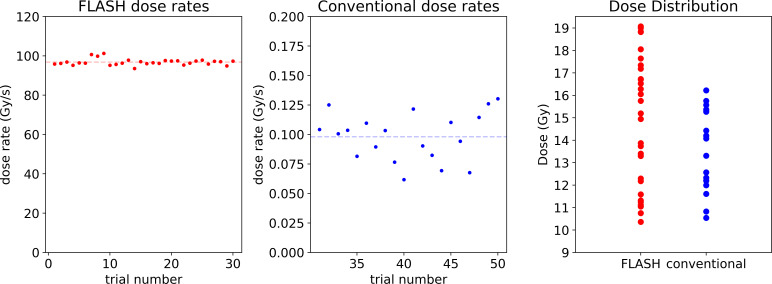
The first 2 panels display the dose rates used for the FLASH and conventional cohorts, respectively. The final panel shows the distribution of doses delivered to the mice in both cohorts. These values are calculated from film measurements taken during the mouse trial and referenced to a calibration run performed separately.

The study made use of nontumor bearing female C57BL/6 mice exclusively, with an average initial weight of 18.77 g. The holder shown in **Figure 1** was built to hold the mice and also connect to the anesthesia line inserted into the slot at the top of the box. Isoflurane gas was mixed with medical air and delivered to a box outside of the cyclotron vault and to the mouse fixture inside the vault. Medical air was used in place of pure oxygen to prevent high tissue oxygenation that might lead to the weakening of any FLASH result. Varying levels of tissue oxygenation are proposed as one of the mechanisms by which the FLASH effect operates [23–26]. Mice were anesthetized shortly before irradiation and held under a continuous flow of anesthesia for the duration of their experimental run. A video feed allowed for monitoring of the mice over the course of the treatment. None of the mice exhibited significant motion. After irradiation, an independent contractor (Charles River Laboratories) managed husbandry and data collection at an offsite facility. This contractor was blind to the dose levels and rates each mouse received. They were also responsible for attaining the approval of an ethical committee for the purposes of this study.

**Figure 2.**
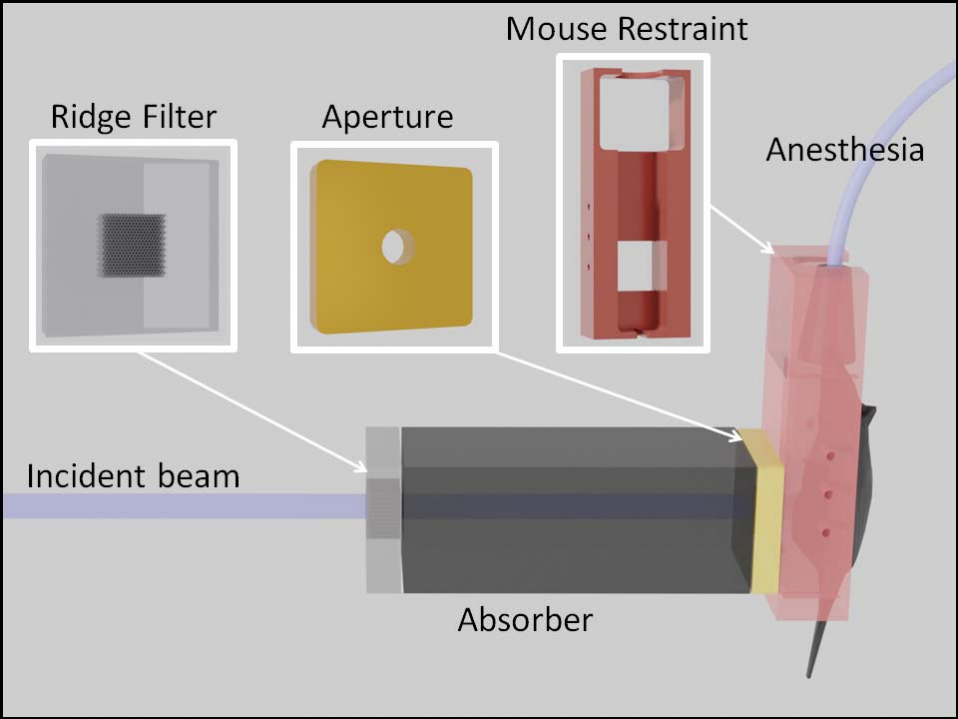
A block diagram of the experimental setup. This representation is not to scale.

**Figure 3.**
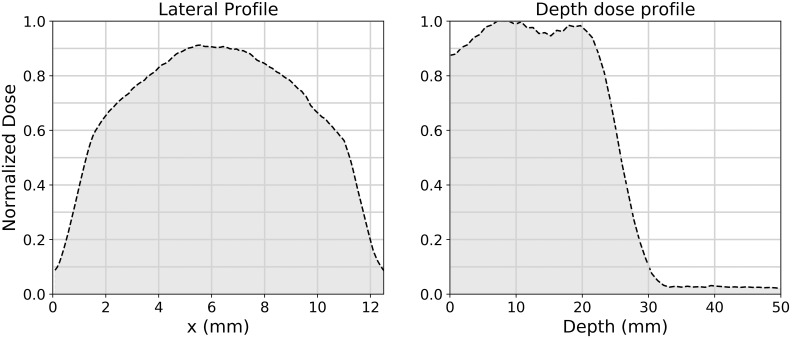
The recorded dose distribution laterally and in depth was recorded with films and a multilayer ion chamber. With maximum dose rates well over 100 Gy/s, the entire irradiated volume receives FLASH dose rates.

## Results

**Figure 4.**
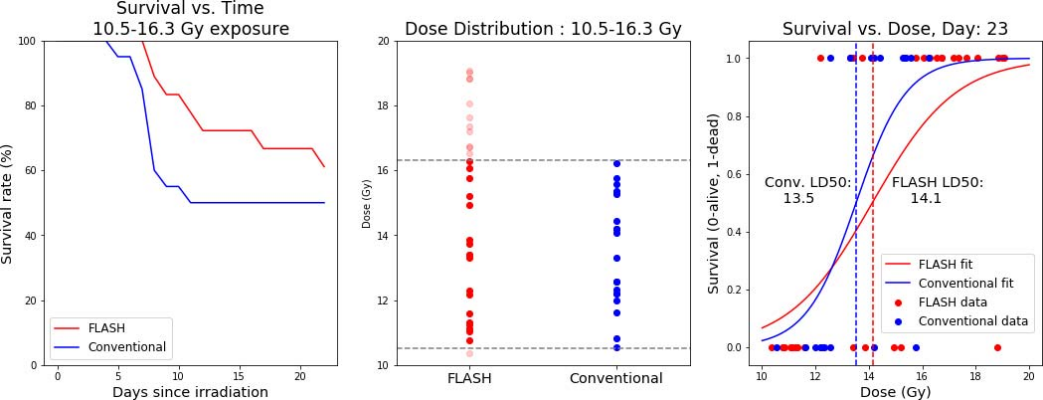
The first plot shows the survival over time of the FLASH and conventional cohorts, with the second plot showing the range of mice used in each cohort for that calculation. If we consider the range of doses spanned by the conventional dose rate group, we find that over the same range of doses, the FLASH cohort had a higher or equivalent rate of survival over the entire range of recorded dates. The averages of the FLASH and conventional cohorts over this range are 13.04 and 13.53 Gy, respectively. The separation observed corresponds to P < .05. In the final plot, a logistic curve was fit to the survivorship information and LD50 values were calculated for the FLASH and conventional cohorts. The results show a separation between the 2 groups, with FLASH mice more likely to survive at higher dose rates than the conventional mice. The calculated LD50 values for the FLASH and conventional cohorts were 14.1 and 13.5, respectively. This amounts to a 19% maximum increase in chance of survival at specific total dose values.

Results of this experiment were recorded with 2 metrics, body weight and mortality. Body weights were recorded daily and any mice that lost more than 20% of their initial weight were euthanized. This was the primary mode of death for the mice in the study. **Figure 4** displays the raw survival information. As shown in **Figure 1**, the distribution of doses delivered was wider in the case of the FLASH cohort than the conventional cohort. The built-in logistic regression class in the Scikit Python package allowed us to fit a curve to the observed survival data. The model employed is of the form:

 *(1)*

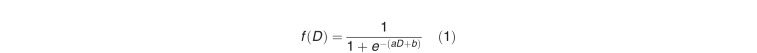




The 2 parameters, *a* and *b*, determined the shape of the curve, with the LD50 represented as –*b*/*a*. These fits show that the FLASH delivery method improves the survivability of the treatment at higher doses. The magnitude of the effect is 19%. The separation of the LD50 values for the 2 cohorts was 0.6 Gy.

**Figure 5.**
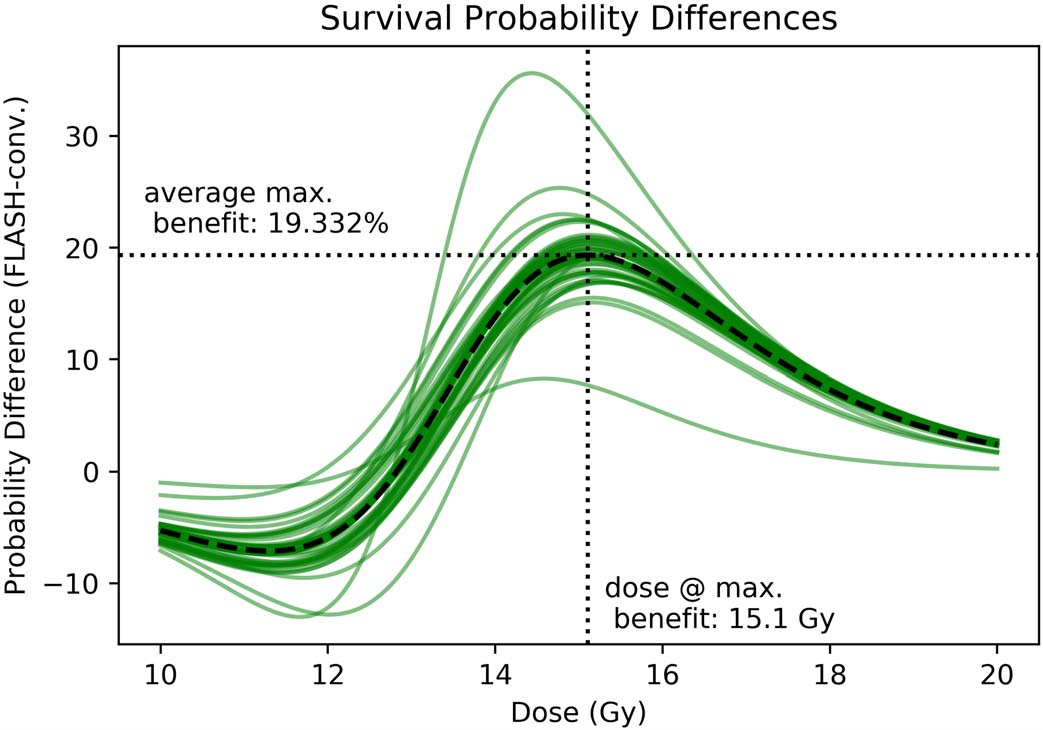
Taking the difference of the fitted chance of survival for the FLASH cohort versus the conventional cohort at all doses, we find the shown curves. Each curve represents the fitting after the removal of one mouse from the study. The variation of the curves under this regime serves as a metric for the significance of the difference in survivability between the 2 cohorts. The black dashed curve shows the average difference between the 2 cohorts, with the maximum variation marked by the dotted lines. The most variation is apparent at higher doses, past the LD50 value of the conventional mouse group. The effect has a maximum of 19% at 15.1 Gy.

The results of this experiment show superior normal tissue sparing for FLASH versus conventional dose rates that is robust to any single mouse being removed from the experiment. From **Figure 5**, that difference is greatest around 15.1 Gy, with an apparent maximum difference of slightly less than 20 percentage points in the likelihood of survival. This is an increase from 65.4 % to 84.7 % survival probability. **Figure 5** does show 2 curves that seem to stand as outliers as compared with the rest. These curves correspond to mice at either end of the dose distribution that can strongly influence the fitting. To establish the reliability of our result, we analyzed the dependence of the separation between the FLASH and conventional cohorts on any given pair of mice. We iteratively removed 1 mouse from each data set and then recomputed the fitting to the logistic curve. The separation between the 2 curves at all dose levels was then recomputed and is represented in **Figure 5**. To evaluate the robustness of the result to fitting routine, we also performed a fit to the survivorship data using the curve_fit routine provided by the SciPy Optimize module. This model was fit to the survivorship data for multiple time points in the study, starting after the first mice began to die. The results of this analysis can be found in the supplementary materials.

## Discussion

Accounting for a relative biologic effectiveness factor for protons on the order of 1.1 to 1.3 [12], our result for the conventional LD50 value matches well with the 14.7 Gy value quoted for electron beams [3]. The “crossover” of the survivorship curves and the apparent centering of the maximal FLASH effect around a particular dose value merit some further discussion. We believe that the crossover in the fits to the FLASH and conventional cohort survivorship curves are mostly likely results of an underconstrained fit to the data rather than a true biologic effect. This study was focused primarily on establishing the LD50 value itself, which is achievable with a relatively small dataset. A higher fidelity matching of the slope of the survivorship curve would be accomplished with a larger number of mice or a more complex mathematic model for the shape of the survivorship curve that reflects the specific biology of the FLASH effect. In the absence of a clear biologic mechanism for the proton FLASH effect, we felt that a simple model was most appropriate. The minimum dose threshold for the FLASH effect has been discussed before but is not yet well understood, particularly in the case of proton deliveries. One possible biologic explanation, if we take for granted the oxygen depletion explanation of the FLASH, is that a sufficiently high dose is required to “use up” all of the oxygen species in the treated region after which point any more doses delivered within a sufficiently short timeline accrues smaller proportional damage to healthy tissue. Choosing a dose much higher than the optimal value eventually does irreparable damage irrespective of the dose rate at which it is delivered, leading to necrosis and death.

The maximal difference in survival between the FLASH and conventional cohorts was approximately 20%. We believe a larger effect would be observable with a different choice of endpoint for the euthanization of the mice. In particular, we have found in discussion with other research groups that similar studies tend to use a more complex metric to decide when moribund sacrifice is required, allowing the bodyweights of the mice to go under 80% of the original value and focusing instead on the behavior of the mice as an indicator of health. In other studies, mice have been seen to recover even after reaching 70% of their original weight. It has been noted that FLASH-treated mice will sometimes drop to lower weights faster before recovering to their original weights, while the health of the conventionally treated mice continues to deteriorate. This being the case, a more comprehensive scoring system for moribund sacrifice of the mice could lead to a significantly larger observed separation. Unfortunately, using such a metric was not feasible for the present study. An LD50 separation of 19% was observed by Loo et al [16] in electrons. There is some indication that an earlier calculation of the LD50 value would have yielded a larger LD50 separation for the present study. This will be considered in future studies.

Future research will include continued improvements to the experimental apparatus for convenience and ease of analysis. In particular, the drift and saturation effects can be addressed in the hardware and electronics to bring down the 5% to 6% dose control error associated with each delivery. While they can be adjusted for in postprocessing by way of the film analysis, it would be preferable to have control hardware capable of higher accuracy. Fortunately, the setup used in this study was more than sufficient to provide well-overlapped dose distributions and to allow subsequent analysis of shifted LD50 values. Changes would include a preamplification stage for the FLASH TIC with a higher saturation threshold. Drift may also be addressed through better electrical isolation of the FLASH TIC itself and a more flexible “on the fly” adjustment for observed drift rates during the experiment. Sufficiently improved dosimetry hardware would allow us to move away from the radiographic film as the primary observable, greatly increasing convenience and decreasing analysis time.

These preliminary results support the existence of a FLASH effect for a proton beam delivered within a SOBP. This suggests the possibility of conformal intensity modulated proton therapy plans combined with the FLASH effect to more effectively protect healthy tissue while maintaining tumor control. In addition to shedding light on the effectiveness of the FLASH effect at the Bragg peak, this study has also precipitated the development of a reliable FLASH dosimetry system that will support further research and testing. This experiment considered a particular delivery regime, with a set target volume and pulse structure. Future research will consider changes to the delivery methodology and pursue the potential for larger treated fields. It is also proposed that other metrics for normal tissue sparing, including microscopic effects reflected in biopsy specimens of treated mice might show a clearer effect for the same conditions.
