# Supplementary figures and images for: Demonstration of the FLASH Effect Within the Spread-out Bragg Peak After Abdominal Irradiation of Mice
Source: Int J Part Ther. 2021 Aug 19;8(4):68–75. doi: 10.14338/IJPT-20-00095 (PMC9009457; doi:10.14338/IJPT-20-00095)

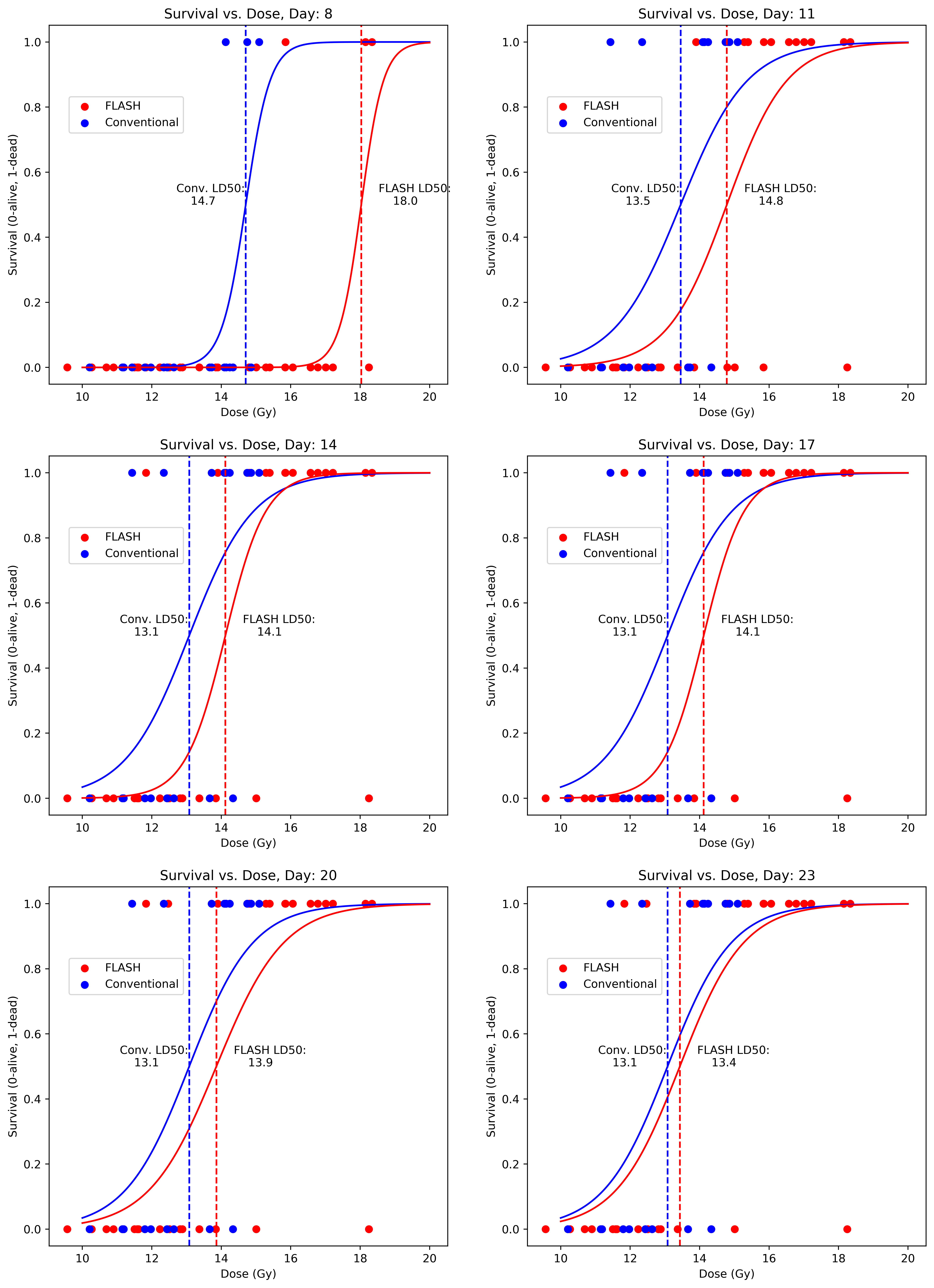

Supplement: Supplementary file 1 [file ijpt-08-04-01_s01.png]
